# Knockdown of lncRNA TUG1 inhibits hippocampal neuronal apoptosis and participates in aerobic exercise-alleviated vascular cognitive impairment

**DOI:** 10.1186/s40659-020-00320-4

**Published:** 2020-11-19

**Authors:** Jing Wang, Yali Niu, Huaying Tao, Mina Xue, Chunxiao Wan

**Affiliations:** 1 Department of Physical and Rehabilitation Medicine, Tianjin Medical University General Hospital, No. 154 Anshan Road, Peace District, Tianjin, 300070 People’s Republic of China; 2 Department of Neurophysiology, Tianjin Medical University General Hospital, Tianjin, 300070 China; 3 Department of Medical Imaging, Tianjin Medical University General Hospital, Tianjin, 300070 China

**Keywords:** Vascular cognitive impairment, lncRNA TUG1, BDNF, Aerobic exercise

## Abstract

**Objectives:**

Our previous study indicated that aerobic exercise relieves cognitive impairment in patients with vascular cognitive impairment (VCI) via regulating brain-derived neurotrophic factor (BDNF), but the mechanism is not yet clear. This study aimed to explore whether lncRNA taurine upregulated gene 1 (TUG1) participates in the process of VCI by regulating BDNF.

**Methods:**

The expressions of TUG1 and BDNF in the serum of VCI patients were detected. The potential molecular mechanisms of TUG1 in regulating hippocampal neuronal apoptosis were explored in oxygen and glucose deprivation-induced (OGD-induced) hippocampal cell line HT22. The VCI mouse model was established, and TUG1 and BDNF were overexpressed via lentivirus injection. The cognitive impairment of mice was detected by the Morris water maze experiment after the aerobic exercise.

**Results:**

The level of TUG1 was elevated in the serum of VCI patients compared with the control group. The knockdown of TUG1 in OGD-induced HT22 cells increased BDNF level and decreased cell apoptosis, and the downregulation of BDNF restored the decreased cell apoptosis. RNA immunoprecipitation and RNA pull-down assays showed that TUG1 could bind to BDNF protein. The aerobic exercise alleviated cognitive impairment and inhibited hippocampal apoptosis in VCI mice. Meanwhile, the overexpression of TUG1 reversed the therapeutic effects of aerobic exercise on cognitive impairment.

**Conclusions:**

The knockdown of TUG1 reduced hippocampal neuronal apoptosis and participates in the aerobic exercise-alleviated VCI, which was partly through regulating BDNF.

## Introduction

Vascular cognitive impairment (VCI) is a cognitive impairment caused by vascular diseases, including mild cognitive impairment and dementia [1]. So far, the pathogenesis of VCI is not clear, and there is no effective treatment, which can only be prevented via treating vascular diseases and other VCI risk factors [1, 2]. Studies have revealed that aerobic exercise improves cardiovascular function in adults and also improves cognitive performance in patients with VCI [3–5]. Nevertheless, the mechanism by which aerobic exercise relieves VCI is unclear.

Long non-coding RNAs (lncRNAs) are widely involved in various human pathological processes, including cerebrovascular diseases [6]. For example, lncRNA SNHG1 and lncRNA MALAT1 have been identified to play a neuroprotective role in ischemic stroke [7, 8]. LncRNA taurine upregulated gene 1 (TUG1) is one of the earliest identified lncRNAs associated with human diseases, which plays a vital regulatory role in various diseases by regulating biological processes, such as cell proliferation, differentiation, and apoptosis [9–11]. Studies have shown that TUG1 may be related to the pathology of neurodegenerative diseases [12]. Nevertheless, it is not explicit whether TUG1 is involved in the pathology of VCI.

Brain-derived neurotrophic factor (BDNF), an important member of the family of neurotrophic factors, is essential for the development of the nervous system and is closely related to neuropathology [13, 14]. We have found that aerobic exercise can alleviate VCI through BDNF [15]. Moreover, we found that TUG1 and BDNF protein may have binding sites through bioinformatics, suggesting that there may be an interaction between TUG1 and BDNF.

This current study aimed to explore whether TUG1 could participate in the pathological process of VCI through regulating BDNF and participate in the process of aerobic exercise-alleviated VCI.

## Materials and methods

### Serum specimens

Fasting venous blood 4 mL was collected from 20 healthy and 20 VCI patients at 9–11 a.m., and serum was collected by centrifugation and stored at − 80 °C for later use. It was found no significant difference in the basic characteristics of the involved patients (p > 0.05, Table 1). All VCI patients and healthy volunteers have signed informed consent, and all human subjects were obtained following ethics committee guidelines and regulations of Tianjin Medical University General Hospital.

**Table 1:** Basic characteristics of the patients involved in this study

Groups	Sex (n)		Age (mean ± SD, year)	BMI (mean ± SD, kg/m^2^)
	Male	Female		
Healthy (n = 20)	11 (55%)	9 (45%)	66.37 ± 7.29	23.09 ± 1.50
VCI (n = 20)	10 (50%)	10 (50%)	65.69 ± 8.17	23.51 ± 0.97

*VCI* vascular cognitive impairment, *SD* standard deviation, *BMI*, body mass index

### ELISA analysis

The blood was natural coagulation at room temperature for 20 min. The supernatant was collected by centrifugation at 1000*g* at 4 °C for 10 min. BDNF concentration was detected using the Human BDNF ELISA KIT (Solarbio, Beijing, China) according to the manufacturer’s instructions.

### Cell culture and treatment

Mouse hippocampal cell line HT22 was purchased from ATCC (Manassas, VA, USA). HT22 cells were cultured in Dulbecco’s modified eagle medium (DMEM; Gibco, New York, USA) mixed with 1% penicillin–streptomycin (Gibco) and 10% fetal bovine serum (FBS). The cells were maintained in a humidified incubator with 95% air and 5% CO_2_ atmosphere at 37 °C.

Oxygen and glucose deprivation (OGD) treatment was used to induce the ischemic injury of HT22 cells. The cell culture medium was changed to glucose-free DMEM (Gibco) and the cells were incubated in a 95% N_2_ and 5% CO_2_ incubator at 37 °C for 1 h. The cell culture medium was replaced with a normal medium containing FBS, and the cells were then cultured at 37 °C with air (21% O_2_) and 5% CO_2_ for 24 h.

### Cell transfection

The sh-TUG1, sh-BDNF, and control shRNA (GenePharma) were constructed into a lentiviral vector and then packaged into lentiviral particles (GenePharma). HT22 cells were infected with the lentivirus (5 × 10^5^ TU per 24-well plate) for 72 h before OGD treatment. The shRNA sequences were shown as follows:

sh-NC: sense 5′-UUCUCCGAACGUGUCACGUTT-3′, antisense 3′-ACGUGACAC GUUCGGAGAATT-5′.

sh-TUG1: sense 5′-CCAUCUCACAAGGCUUCAATT-3′, antisense 3′-TTGGUAG AGUGUUCCGAAGUU-5′.

sh-BDNF: sense 5′-GGTGATGCTCAGCAGTCAAGT-3′, antisense 3′-CCACTACG AGTCGTCAGTTCA-5′.

### Quantitative real-time PCR (qRT-PCR)

The total RNA of serum, mouse hippocampus tissue, or HT22 cells was isolated using Trizol reagent (Invitrogen, Carlsbad, CA, USA). cDNA was synthesized by the PrimeScript RT Master Mix (Takara, Dalian, China) with 0.1 µg of total RNA. The levels of TUG1 and BDNF mRNA was detected by the SYBR Premix Ex Taq (TaKaRa) and ABI 7500 Real-Time PCR system (Applied Biosystems, Carlsbad, USA). β-actin was used as an internal control. The qRT-PCR primers were shown as follows:

TUG1: (F) 5′-CTGAAGAAAGGCAACATC-3′, (R) 5′-GTAGGCTACTACAGGAT TTG-3′.

BDNF mRNA: (F) 5′-AGGACAGCAAAGCCACAATGTTCC-3′, (R) 5′-TGGACG TTTGCTTCTTTCATGGGC-3′.

β-actin: (F) 5′-TGAGAGGGAAATCGTGCGTGAC-3′, (R) 5′-AAGAAGGAAGGC TGGAAAAGAG-3′.

### Western Blot

HT22 cells or mouse hippocampus tissue were lysed using RIPA lysis buffer (Beyotime, Shanghai, China). The BCA Kit (Beyotime) was used to detect protein concentration. Equal amounts of protein lysate were used for SDS-PAGE and transferred the protein to the PVDF membrane (Millipore, Bedford, USA) via electro-transfer [16]. Then, the PVDF membrane was blocked using QuickBlock™ Blocking Buffer (Beyotime) and incubated overnight with the Anti-BDNF antibody (ab108319,1:1000) and the Anti-β-actin antibody (ab8227, 1:1000). After that, the second antibody was incubated for 3 h at 4 °C. The membranes were detected using the ECL system (Beyotime). β-actin was used as a control for total protein amount.

### Flow cytometry

HT22 cells or slices of mouse hippocampal tissues were detected apoptosis using the Annexin V-FITC Apoptosis Detection Kit (Solarbio Life Sciences, Beijing, China) according to the manufacturer’s instructions. Briefly, 3 mL Binding Buffer (10×) was diluted to 30 mL with 27 mL of deionized water. Cells (1 × 10^6^) were collected and washed with cold phosphate buffer saline (PBS). Then, cells were suspended in 1 mL 1× Binding Buffer, followed by the centrifugation at 300×*g* for 10 min before discarding the supernatant. Cells were resuspended with 1 mL 1× Binding Buffer to make the cell density reach 1 × 10^6^ cells/mL. Cells with 100 µL (1 × 10^5^) were added to each tube. Then, 5 µL Annexin V-FITC was added to the tube for the mixture of 10 min at room temperature in dark. After that, 5 µL PI was added and the cells were cultured for 5 min at room temperature in dark. After being mixed with 500 µL PBS, the cells were applied to detect apoptosis using the flow cytometer (FACSCanto II; BD Biosciences, San Jose, CA, USA).

### Cell viability assay

Cell viability was detected using the MTT (3-(4, 5-dimethylthiazol-2-yl)-2, 5-diphenyl-2H-tetrazol-3-ium bromide) Cell Proliferation and Cytotoxicity Assay Kit (Solarbio Life Sciences, Beijing, China). In brief, the log phase cells were collected and the cell suspension concentration was adjusted. Then, the cells were seeded into 96-well plates with 180 µL per well (3000–10,000 cells/well). The cells were placed in a 37 °C, 5% CO_2_ incubator for 6–24 h. After carefully aspirating the supernatant, we added 90 µL of fresh culture medium, followed by adding 10 µL of MTT solution and continued to incubate cells for 4 h. Then, 110 µL of Formazan dissolving solution was added to each well and the cells were placed on a shaker at low speed for 10 min. The absorbance of each well at 490 nm was measured in an enzyme-linked immunoassay instrument (JK-MR-5031, JK Imaging, CA, USA).

### RNA pull-down assay

The biotinylated DNA probe complementary of TUG1 (sense) and biotin-labeled antisense TUG1 (anti-sense) were synthesized (Genepharma). Sense or anti-sense was purified and transfected into HT22 cells. After 48 h, the cells were collected. RNA pull-down assays were performed using the Pierce Magnetic RNA-Protein Pull-Down Kit (Thermo Scientific, Waltham, MA, USA) according to the manufacturer’s instructions. The level of BDNF in the complex was detected via Western Blot.

### RNA immunoprecipitation (RIP) assay

HT22 cells were collected using RIPA lysis buffer (Beyotime). RIP assay was performed using the Magna RIP RNA-Binding Protein Immunoprecipitation Kit (Millipore) and anti-BDNF or IgG (Millipore). The level of TUG1 in the complex was detected via qRT-PCR.

### Animals

C57BL/6 male mice (6–7 weeks old) were purchased from the Charles River (Beijing, China), and were kept in light/dark cycle for 12 h at 22–25 °C for free feeding. All animal experiments used in this study were approved by the Animal Ethical Committee of the Tianjin Medical University General Hospital.

### VCI mouse model

The VCI mouse model (the 2VO group) was established by ligating bilateral common carotid arteries (CCAs) according to the previous description [17]. In brief, mice (n = 6) were anesthetized with 10% chloral hydrate (400 mg/kg, intraperitoneal injection). The bilateral CCAs were ligated with 4−0 silk sutures for 20 min. The sutures were removed and the bilateral CCAs were visually inspected for reperfusion at the end of the occlusion period. The bilateral CCAs were again ligated for 20 min after the blood flow was restored for 10 min. The occlusion and the reperfusion were repeated three times. Bilateral CCAs of mice in the Sham group were not ligated.

Lentiviral TUG1 overexpression vector (Lenti-TUG1) and lentiviral vector (Lenti-control) containing a sequence of scrambled control were designed, packaged, and purified by GenePharma (Shanghai, China). Mice were injected with Lenti-TUG1 or Lenti-control via bilateral ventricles. Mice underwent aerobic exercise via an animal treadmill and mice performed 30 min (12 m/min) treadmill exercise every day (5 days a week) for 4 weeks [18]. The Morris water maze experiment was then performed. After the Morris water maze experiment, mice were sacrificed, and the brains were collected. Hippocampal tissues were quickly separated on ice, and the tissues were made into 10% tissue homogenate for further experiments.

### The Morris water maze experiment

The Morris water maze was used to assess cognitive impairment in mice using the previously described method [19, 20]. In brief, the circular pool was filled with about 25 °C of water and maintain the temperature. Then, the mice were trained for 4 days to find the platform. After that, skimmed milk powder was added to make the water opaque. The time at which mice found the platform was recorded, and if it exceeded 60 s, it was recorded as 60 s. Performing five times a day for a total of 5 days. After the Morris water maze was completed, the mouse was sacrificed and hippocampal tissue was collected for subsequent studies.

### Statistical analysis

Statistics were calculated via SPSS 22.0 (Chicago, IL, USA), and all data were presented as mean ± standard deviation (SD). The difference among multiple groups was compared via the one-way analysis of variance (ANOVA) followed by the LSD post hoc test. The difference between the two groups was compared via the Student’s t-test. The *P*-value < 0.05 was considered statistically significant.

## Results

### Aberrant expression of TUG1 and BDNF in the serum of VCI patients

**Fig. 1:**
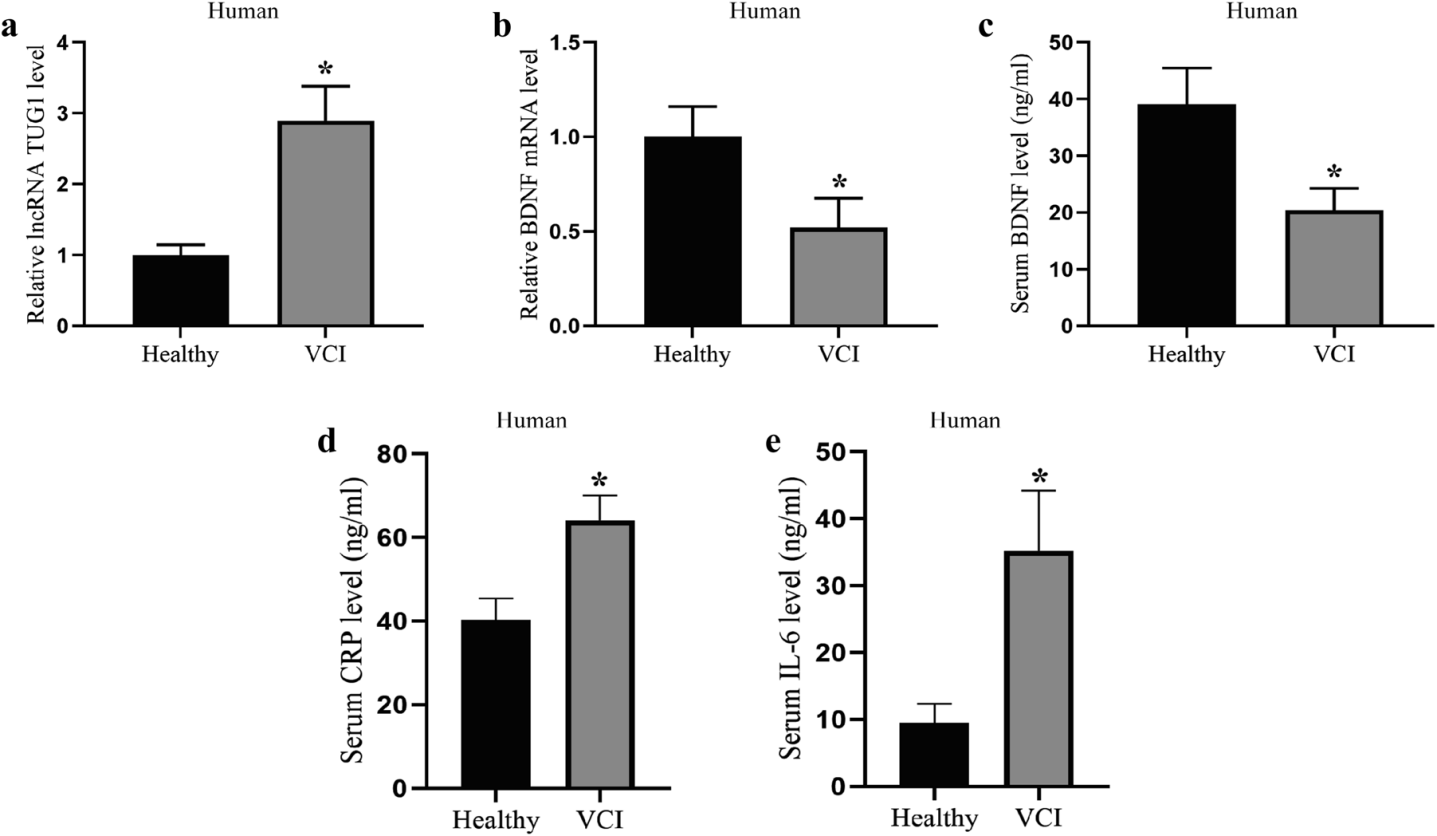
Aberrant expression of TUG1 and BDNF in the serum of VCI patients. The 4 mL of fasting venous blood was collected from healthy people (n = 20, the Healthy group) and VCI patients (n = 20, the VCI group), and serum was collected by centrifugation. **a** The TUG1 level was detected by qRT-PCR. **b** The BDNF mRNA level was detected by qRT-PCR. The serum BDNF level (**c**), CRP level (**d**), and IL-6 level (**e**) were detected by ELISA. *P < 0.05 vs. the Healthy group

To investigate whether TUG1 and BDNF were differentially expressed during VCI, we detected the levels of TUG1 and BDNF in the serum of healthy people (n = 20) and VCI patients (n = 20). The results of qRT-PCR showed that compared with healthy people, the level of TUG1 was observably elevated in the serum of VCI patients, which was 2.9 times higher than that in healthy people (Fig. 1a), while the mRNA level of BDNF was 0.52 times lower than that in healthy people (Fig. 1b). Meanwhile, ELISA results showed that serum BDNF levels were markedly reduced in VCI patients (Fig. 1c). Besides, the serum CRP (Fig. 1d) and IL-6 (Fig. 1e) levels in the VCI patients were upregulated in comparison with the healthy controls, indicating the brain injury of the VCI patients. These results indicated that TUG1 and BDNF may be involved in the pathological process of VCI.

### TUG1 knockdown reduces the apoptosis of OGD-induced hippocampal neurons

**Fig. 2:**
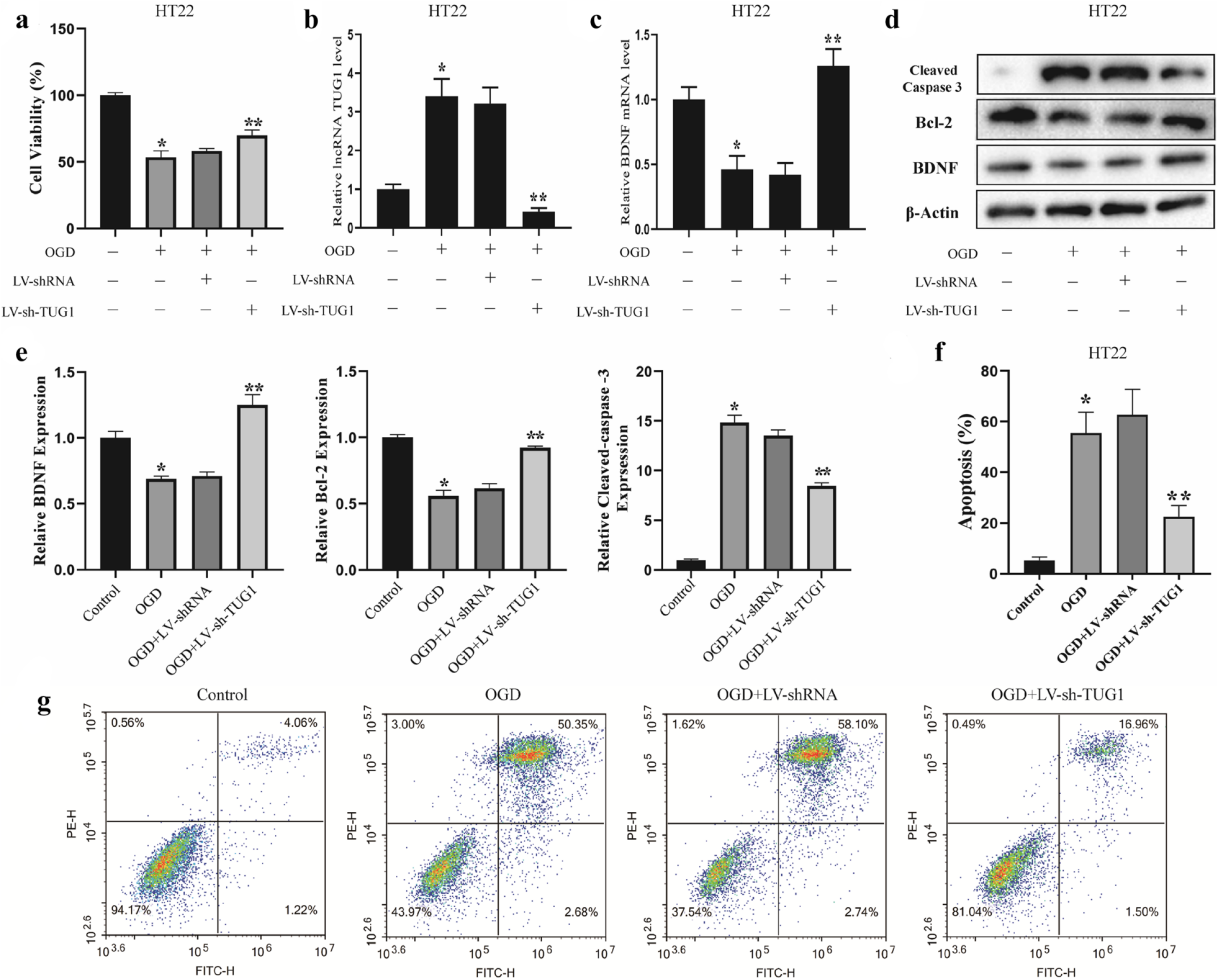
TUG1 knockdown reduces the apoptosis of OGD-induced hippocampal neurons. Murine hippocampal neuron cell line HT22 cells were divided into four groups: the Control group (normally cultured cells), the OGD group (cells were reoxygenated for 24 h after OGD treatment for 1 h), the OGD + LV-shRNA group (cells were transfected with LV-shRNA for 72 h before the OGD treatment), and the OGD + LV-sh-TUG1 group (cells were transfected with LV-sh-TUG1 for 72 h before the OGD treatment). **a** The cell viability was detected by MTT assay. **b** The TUG1 level was detected by qRT-PCR. **c** The BDNF mRNA level was detected by qRT-PCR. **d, e** The protein expressions of BDNF, cleaved-Caspase 3, and Bcl-2 were detected by western blotting. **f**, **g** Cell apoptosis was detected by flow cytometry. The experiments were repeated three times (n = 3). *P < 0.05 vs. the control group; **P < 0.05 vs. the OGD + LV-shRNA group

Murine hippocampal neuron cell line HT22 was treated with OGD to mimic VCI in vitro [15]. After the OGD treatment, the cell viability of HT22 cells was inhibited, which was partly reversed by the lentivirus-mediated TUG1 knockdown (Fig. 2a). In the OGD-treated HT22 cells, the level of TUG1 was increased (Fig. 2b), while the expression level of BDNF was decreased at mRNA levels (Fig. 2c), and these effects were negated by the lentivirus-mediated TUG1 knockdown. The cell apoptosis was elevated in OGD-induced HT22 cells with the increased expression of the pro-apoptotic marker (cleaved caspase 3) and the decreased expression of the anti-apoptotic marker (Bcl2) (Fig. 2d–g). Such promotive effects mediated by the OGD treatment were reversed by the lentivirus-mediated TUG1 knockdown (Fig. 2d–g). These results suggested that TUG1 may regulate cell apoptosis in VCI.

### TUG1 knockdown reduces hippocampal neuronal apoptosis by upregulating BDNF

**Fig. 3:**
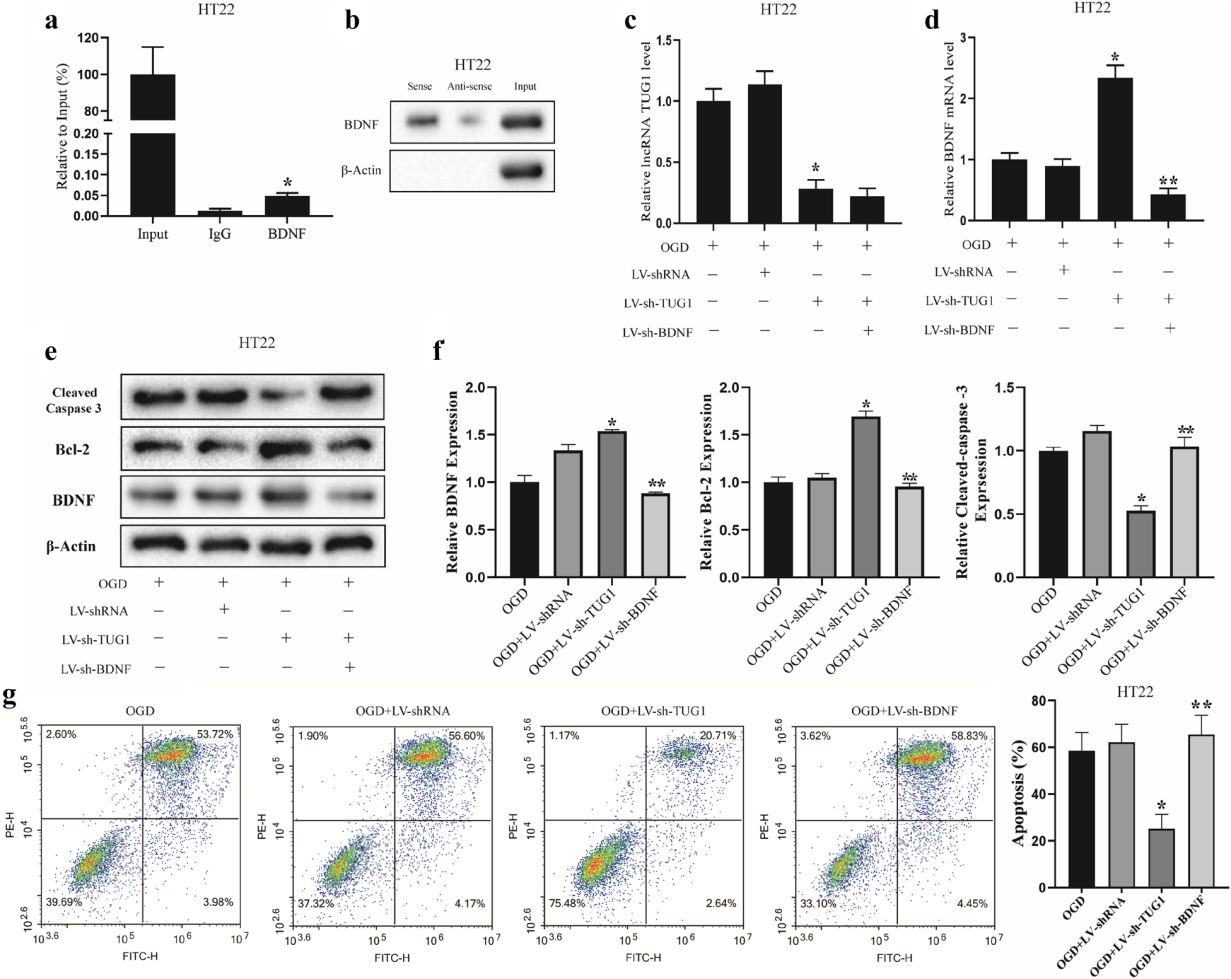
TUG1 knockdown reduces neuronal apoptosis by upregulating BDNF. **a** RIP results showed that TUG1 was enriched in the anti-BDNF immunoprecipitants. *P < 0.05 vs. the IgG group. **b** BDNF was enriched in the TUG1-pulled down compounds. HT22 cells were divided into four groups, the OGD group (cells were reoxygenated for 24 h after OGD treatment for 1 h), the OGD + LV-shRNA group (cells were transfected with LV-shRNA for 72 h before the OGD treatment), the OGD + LV-sh-TUG1 group (cells were transfected with LV-sh-TUG1 for 72 h before the OGD treatment), and the OGD + LV-sh-BDNF group (cells were transfected with LV-sh-BDNF for 72 h before the OGD treatment). **c** The TUG1 level was detected by qRT-PCR. **d** The BDNF mRNA level was detected by qRT-PCR. **e**, **f** The protein expressions of BDNF, cleaved-Caspase 3, and Bcl-2 were detected by western blotting. **g** Cell apoptosis was detected by flow cytometry. The experiments were repeated three times (n = 3). *P < 0.05 vs. the OGD + LV-shRNA group; **P < 0.05 vs. the OGD + LV-sh-TUG1 group

To further explore the relationship between TUG1 and BDNF, we detected whether TUG1 binds to BDNF via RIP and RNA pull-down analysis. RIP results showed that TUG1 was enriched in the anti-BDNF immunoprecipitants (Fig. 3a), and BDNF was enriched in the TUG1-pulled down compounds (Fig. 3b). These results indicated that TUG1 could bind to BDNF protein. Moreover, under the OGD treatment, the knockdown of TUG1 elevated mRNA and protein levels of BDNF, and the lentivirus-mediated BDNF knockdown reversed such effect (Fig. 3c, d). Furthermore, the lentivirus-mediated BDNF knockdown reduced the elevation of cell apoptosis which was caused by TUG1 knockdown in OGD-induced HT22 cells (Fig. 3e–g). The trends of protein expressions of cleaved-Caspase 3 and Bcl-2 were consistent with the apoptotic results (Fig. 3e–g). These results indicated that TUG1 negatively regulates BDNF expression by binding to BDNF, thus affecting the apoptosis of OGD-induced hippocampal neurons.

### Aerobic exercise alleviates mouse VCI via downregulating TUG1

**Fig. 4:**
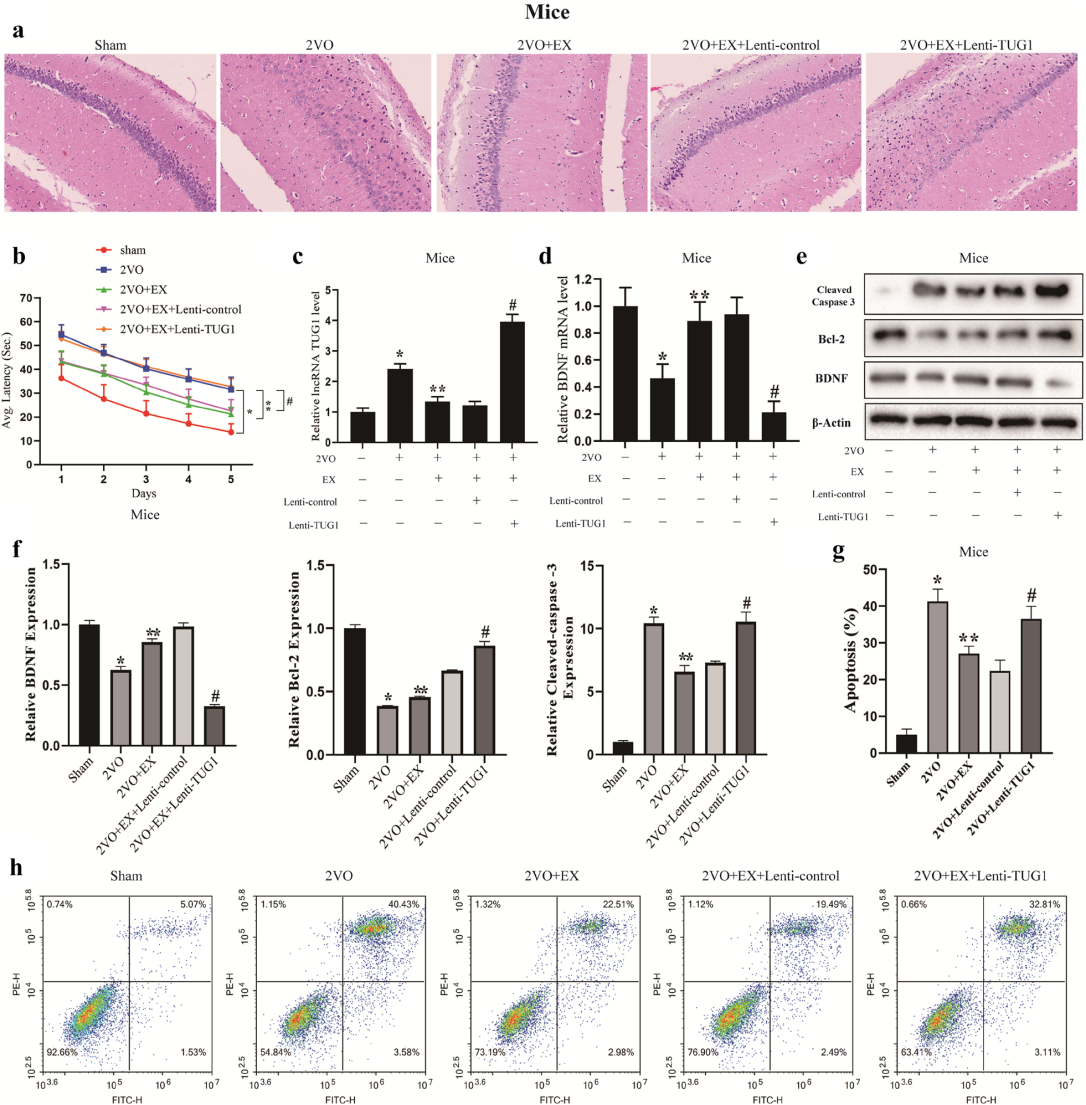
Aerobic exercise alleviates mouse VCI via downregulating TUG1. The mice were divided into five groups (n = 6 in each group), the sham group, the 2VO group (VCI mouse model was established by ligating bilateral CCAs), the 2VO + EX group (VCI mice underwent aerobic exercise for 4 weeks), the 2VO + EX + Lenti-control group (Lenti-control were injected into the ventricle of VCI mice, followed by the aerobic exercise for 4 weeks) and the 2VO + EX + Lenti-TUG1 group (Lenti-TUG1 were injected into the ventricle of VCI mice, followed by the aerobic exercise for 4 weeks). **a** Mouse brain tissues were collected and were stained by H&E. **b** The Morris water maze test was used to detect the latency for mice to reach the platform. **c** The TUG1 level in hippocampal tissues was detected by qRT-PCR. **d** The BDNF mRNA level was detected by qRT-PCR. **e**, **f** The protein expressions of BDNF, cleaved-Caspase 3, and Bcl-2 were detected by western blotting. **g**, **h** Cell apoptosis was detected by flow cytometry. *P < 0.05 vs. the sham group; **P < 0.05 vs. the 2VO group; ^#^P < 0.05 vs. the 2VO + EX + Lenti-control group

To further explore whether TUG1 participates in the therapeutic effect of aerobic exercise on VCI, Lenti-control or Lenti-TUG1 were injected into the ventricle of the VCI mice, followed by the aerobic exercise for 4 weeks (n = 6 in each group). The H&E staining results showed that the brain tissues in the 2VO group presented hypoxic regions, while after the aerobic exercise for 4 weeks, the hypoxic regions were reduced. Besides, the overexpression of TUG1 negated the effect of the aerobic exercise (Fig. 4a). The Morris water maze test is widely used to detect spatial learning and memory in laboratory mice [21]. We used the Morris water maze test to evaluate the effects of TUG1 on the cognitive function of VCI mice. The results revealed that the latency to reach the platform was increased in the VCI mice compared with the sham mice, but after the aerobic exercise, the latency was reduced and such reduction was restored by the Lenti-TUG1 injection (Fig. 4b). Next, we collected hippocampal tissues, and the level of TUG1 in VCI mice was restored by the Lenti-TUG1 injection, which was reduced after the aerobic exercise (Fig. 4c). The Lenti-TUG1 injection also reduced the enhancement of the BDNF expression that was raised after the aerobic exercise (Fig. 4d). Besides, the Lenti-TUG1 injection restored the reduction of the apoptosis which was reduced after the aerobic exercise (Fig. 4e–h). The trends of protein expressions of cleaved-Caspase 3 and Bcl-2 were consistent with the apoptotic results (Fig. 4e–h). These findings indicated that aerobic exercise alleviates mouse VCI partly via downregulating TUG1.

## Discussion

Cognitive impairment of vascular etiology is the leading cause of dementia in East Asia and Western countries following Alzheimer’s disease [22]. Vascular dementia is the most serious form of VCI [23]. Nevertheless, the molecular mechanism of VCI pathogenesis is poorly understood [1, 2]. More and more studies have indicated that aerobic exercise can improve cognitive impairment including VCI [24], but the mechanism is not clear. In our previous study, we have found that aerobic exercise can alleviate VCI through upregulating BDNF [15]. In this study, we found that the expression of TUG1 was significantly elevated in the serum of VCI patients compared with healthy people. Moreover, knockdown of TUG1 inhibits hippocampal neuronal apoptosis by promoting the expression of BDNF in OGD-induced HT22 cells. Finally, we demonstrated that aerobic exercise can improve cognitive impairment and inhibit hippocampal apoptosis of VCI mice by downregulating TUG1 expression. Our findings provide a potential target for the treatment of VCI and provide new insights into the potential mechanism of the aerobic exercise-alleviated VCI.

Cerebral hypoperfusion is generally considered to be the underlying pathophysiological mechanism of VCI [25]. Model animal experiments have shown that cerebral hypoperfusion can cause white matter damage, brain atrophy, and memory impairment [25, 26]. Furthermore, About 30% of survivors with ischemic stroke will develop VCI or vascular dementia [27]. The cerebral hypoperfusion model is usually used as a VCI model. In this study, we established the mouse VCI model by ligating bilateral CCAs to mimic the cerebral hypoperfusion in vivo. The Morris water maze was used to determine the cognitive impairment of the VCI mice. We found that the knockdown of TUG1 can reduce hippocampal neuronal apoptosis in vitro and the overexpression of TUG1 can negate the anti-apoptotic effect of the aerobic exercise on VCI mice in vivo. Studies have shown that apoptosis can explain the neurocyte loss observed in many neurological diseases including VCI [28]. Thus, we used the in vitro model and the in vivo model to demonstrate the effect of TUG1 in hippocampal neuronal apoptosis-mediated VCI.

Many investigations indicate that aerobic exercise improves the function of the central nervous system, the attention, and the working memory, and reduces the risk of cognitive impairment and dementia [14, 29, 30]. Similarly, aerobic exercise can also alleviate VCI [3, 31]. BDNF is the most common neurotrophic protein in the adult brain, and BDNF plays a key role in hippocampal long-term potentiation, which is considered the foundation of learning and memory [32]. Moreover, aerobic exercise can increase the level of BDNF in peripheral blood, which can be absorbed by the brain tissue, thereby protecting the nerve and improving cognition [14, 33, 34]. Our study found that TUG1 can bind to BDNF and negatively regulates its expression. Meanwhile, we found that the overexpression of TUG1 reversed the improvement of the aerobic exercise on the cognitive ability and hippocampal apoptosis of VCI mice through regulating BDNF. These results indicated that the aerobic exercise alleviates VCI in part by regulating TUG1/BDNF.

## Conclusions

In conclusion, our findings indicated that the TUG1/BDNF axis participates in the aerobic exercise-alleviated VCI by reducing hippocampal neuronal apoptosis.

## Data Availability

All data generated or analyzed during this study are included in this published article.

## Abbreviations

VCI: Vascular cognitive impairment
qRT-PCR: Quantitative real-time PCR
TUG1: Taurine upregulated gene 1
OGD: Oxygen and glucose deprivation
BDNF: Brain-derived neurotrophic factor
RIP: RNA immunoprecipitation
CCA: Common carotid arteries
MTT: 3-(4,5-dimethylthiazol-2-yl)-2,5-diphenyl-2H-tetrazol-3-ium bromide
PBS: Phosphate buffer saline
lncRNAs: Long non-coding RNAs
